# Enhanced neutrophil extracellular trap generation in rheumatoid arthritis: analysis of underlying signal transduction pathways and potential diagnostic utility

**DOI:** 10.1186/ar4579

**Published:** 2014-06-13

**Authors:** Chanchal Sur Chowdhury, Stavros Giaglis, Ulrich A Walker, Andreas Buser, Sinuhe Hahn, Paul Hasler

**Affiliations:** 1 Department of Biomedicine, University Hospital Basel, Hebelstrasse 20, 4032 Basel, Switzerland; 2 Department of Rheumatology, Kantonsspital Aarau, Tellstrasse, 5001 Aarau, Switzerland; 3 Department of Rheumatology, University Hospital Basel, Basel, Switzerland; 4 Division of Haematology, Department of Internal Medicine, University Hospital Basel, Blood Transfusion Centre, Swiss Red Cross, Basel, Switzerland

## Abstract

**Introduction:**

Neutrophil extracellular traps (NETs) have recently been implicated in a number of autoimmune conditions, including rheumatoid arthritis (RA). We examined the underlying signaling pathways triggering enhanced NETosis in RA and ascertained whether the products of NETosis had diagnostic implications or usefulness.

**Methods:**

Neutrophils were isolated from RA patients with active disease and from controls. Spontaneous NET formation from RA and control neutrophils was assessed *in vitro* with microscopy and enzyme-linked immunosorbent assay (ELISA) for NETosis-derived products. The analysis of the signal-transduction cascade included reactive oxygen species (ROS) production, myeloperoxidase (MPO), neutrophil elastase (NE), peptidyl arginine deiminase 4 (PAD4), and citrullinated histone 3 (citH3). NET formation was studied in response to serum and synovial fluid and immunoglobulin G (IgG) depleted and reconstituted serum. Serum was analyzed for NETosis-derived products, for which receiver operator characteristic (ROC) curves were calculated.

**Results:**

Neutrophils from RA cases exhibited increased spontaneous NET formation *in vitro*, associated with elevated ROS production, enhanced NE and MPO expression, nuclear translocation of PAD4, PAD4-mediated citrullination of H3, and altered nuclear morphology. NET formation in both anti-citrullinated peptide antibody (ACPA)-positive and -negative RA was abolished by IgG depletion, but restored only with ACPA-positive IgG. NETosis-derived products in RA serum demonstrated diagnostic potential, the ROC area under the curve for cell-free nucleosomes being >97%, with a sensitivity of 91% and a specificity of 92%. No significant difference was observed between ACPA-positive and -negative cases.

**Conclusions:**

Signaling elements associated with the extrusion of NETs are significantly enhanced to promote NETosis in RA compared with healthy controls. NETosis depended on the presence of ACPA in ACPA-positive RA serum. The quantitation of NETosis-derived products, such as cell-free nucleosomes in serum, may be a useful complementary tool to discriminate between healthy controls and RA cases.

## Introduction

A novel feature of the biology of polymorphonuclear granulocytes (PMNs) is their ability to generate neutrophil extracellular traps (NETs) [1] via a distinct process of cell death termed NETosis [2]. NETs consist of extruded chromosomal DNA decorated with granular components that include antimicrobial peptides and proteases. The molecular pathways leading to NETosis encompass calcium mobilization, generation of reactive oxygen species (ROS), nuclear delobulation involving the enzymatic activities of myeloperoxidase (MPO) and neutrophil elastase (NE), and chromatin modification via the citrullination of histones by peptidyl arginine deiminase 4 (PAD4) [2-6].

A number of studies have implicated NETs in the etiology of autoinflammatory or autoimmune conditions such as preeclampsia, Felty syndrome, systemic lupus erythematosus (SLE), multiple sclerosis, and, most recently, rheumatoid arthritis (RA) [7-13]. In the context of RA, these findings are especially interesting, as NETs have been proposed to contribute to the generation of anti-citrullinated protein antibody (ACPA) autoantigens, and may also be a target for autoantibodies [13,14]. PMNs isolated from RA patients showed an increased propensity to undergo spontaneous and LPS-induced NETosis, which was in part mediated by TNF and IL-17 and could be inhibited by blocking NADPH oxidase or PAD4. Whereas the citrullinated autoantigens vimentin and α-enolase were expressed on NETs from RA PMNs, antibodies to the former were able to induce NET formation by healthy control PMNs [13,14].

As we had previously detected significantly increased concentrations of cell-free DNA in the sera of RA patients compared with healthy controls, we were intrigued whether the provenance of this material involved NETosis [15]. The premise for the current investigation was that a link between circulating cell-free DNA levels and NETs has previously been made in a number of conditions, including preeclampsia, sepsis, cancer, thrombosis, or even storage of blood-transfusion products [16-19].

In view of these findings and reports on the complex involvement of neutrophil NETs in autoimmunity, we sought to investigate the NETotic response of PMNs in RA, with particular regard to the underlying signal-transduction cascade, and whether the products of overt NETosis could be diagnostically useful.

## Materials and methods

### Human subjects

All patients fulfilled the American College of Rheumatology classification criteria for RA, or for systemic lupus erythematosus, respectively. Healthy volunteers, matched for gender and age, were recruited at the hospitals or at the Blood Bank of the Swiss Red Cross, Basel. Inclusion criteria for healthy controls were fair general condition, age ≥28 and ≤70 years, and for blood donors fulfilling national criteria for blood donation. Exclusion criteria were current or previous systemic autoimmune disease, asthma and reconvalescence after major illness, surgery, current medication with corticosteroids, immunosuppressive agents, and malignant neoplasia or chemotherapy within 5 years before recruitment for the study. RA cases had a DAS ≥2.6, were from age ≥27 to ≤70 years, and had no other systemic autoimmune disease, including ankylosing spondylitis and psoriatic arthritis.

Exclusion criteria were corticosteroids ≥40 mg equivalent of prednisone daily, and those mentioned earlier for healthy controls. Informed, written consent was obtained from all subjects in the study, which was approved by the Cantonal Ethical Review Boards of Aargau-Solothurn and Basel/Basel-Land, Switzerland.

### Preparation of plasma and serum

Plasma and serum were collected and processed as described previously [15]. Samples were studied immediately or stored at -80°C until analysis.

### Cell isolation

PMNs were isolated with Dextran-Ficoll density centrifugation [8]. Cell viability was 96% to 98%, with a purity of >95% PMNs. Neutrophils seeded in 24-well plates were allowed to settle for 1 hour at 37°C under 5% CO_2_ before further experimentation.

### Cell-free DNA isolation and quantification

Cell-free DNA extracted from 850 μl plasma or serum by using the QIAamp Circulating Nucleic Acid Kit (Qiagen, Valencia, CA, USA) was quantified by TaqMan real-time PCR (StepOne Plus Real-Time PCR System, Applied Biosystems, Foster City, CA, USA) specific for the glyceraldehyde-3-phosphate dehydrogenase (*GAPDH*) gene [15].

### Detection of neutrophil elastase (NE), myeloperoxidase (MPO), and cell-free nucleosomes

The concentrations of neutrophil elastase (NE) and myeloperoxidase (MPO) were measured with sandwich ELISA (Elastase/a1-PI Complex ELISA Kit, Calbiochem/EMD, Gibbstown, NJ, USA) and the human (MPO ELISA Kit, Hycult Biotech, Plymouth Meeting, PA, USA) respectively. Nucleosomes were measured by using the Human Cell Death Detection ELISA^PLUS^ (Roche Diagnostics, Basel, Switzerland). Cell-culture supernatants were incubated with DNaseI (10 U for 5 minutes) (Roche Diagnostics) before analysis [20].

### MPO/DNA complex detection

MPO is present on extruded NETs. To detect such structures, NET-associated MPO/DNA complexes were quantified by using a modified capture ELISA [21]. In brief, NET-associated MPO in serum or culture supernatant was captured by using the coated 96-well plate of the human MPO ELISA Kit, (Hycult Biotech), after which the NET-associated DNA backbone was detected by using the detection antibody of the Human Cell Death Detection ELISA^PLUS^ (Roche Diagnostics).

### PAD4/DNA-complex detection

To detect the presence of PAD4 on extruded NETs in culture supernatants after spontaneous NETosis, cell-free PAD4/DNA complexes were quantified by using a modified capture ELISA, akin to that described for MPO earlier. In brief, cell-free PAD4 was captured by using the coated 96-well plate of a commercial human PAD4 ELISA (USCN Life Science, Inc., Wuhan, China), and associated DNA was detected by using Human Cell Death Detection ELISA^PLUS^ kit (Roche Diagnostics).

### ROS generation analysis

ROS was measured by using a 2′,7′-dichloro dihydrofluorescein diacetate (DCFH-DA) assay [22]. The 5 × 10^5^ cells in a final volume of 500 μl were incubated for 30 minutes with 25 μ*M* DCFH-DA (Sigma-Aldrich, St. Louis, MO, USA). Fluorescence was measured with flow cytometry (FACSCalibur; BD Biosciences, San Jose, CA, USA).

### Fluorescence and scanning electron microscopy

The 5 × 10^4^ cells isolated PMNs seeded on poly-L-lysine-coated coverslips (BD Biosciences) were stimulated with phorbol-12-myristate-13-acetate (PMA, Sigma-Aldrich) for 90 minutes and dehydrated with a graded ethanol series (30%, 50%, 70%, 100%) [8], coated with 2-nm platinum, and analyzed with a Nova NanoSEM 230 scanning electron microscope (FEI Co., Hillsboro, OR, USA). PMNs were incubated for 10 minutes with 5 μ*M* Sytox Green dye (Invitrogen Life Technologies, San Diego, CA, USA) for assessment of NETs with an Axiovert fluorescence microscope (Carl Zeiss) coupled to a Zeiss AxioCam color CCD camera (Carl Zeiss Microimaging, Oberkoch, Germany) [8,23].

### Immunohistochemical staining and quantification of NETs

The 5 × 10^4^ isolated PMNs were seeded on poly-L-lysine-coated glass coverslips (BD Biosciences, San Jose, CA, USA) in tissue-culture wells and allowed to settle before stimulation, as described earlier. Coverslips were rinsed with ice-cold HBSS and the cells fixed with 4% paraformaldehyde and blocked overnight (HBSS with 10% goat serum, 1% BSA, 0.1% Tween20, and 2 m*M* EDTA) at 4°C. NETs were detected with rabbit anti-NE (Abcam, Cambridge, MA, USA), rabbit anti-MPO (Dako, Glostrup, Denmark), two different rabbit anti-PAD 4 (Abcam), mouse anti-PAD4 (Abcam), mouse anti-histone H1 + core proteins (EMD Millipore, Billerica, MA, USA), and rabbit anti-citrullinated histone H3 (citH3, Abcam). Secondary antibodies were goat anti-rabbit IgG AF555, goat anti-rabbit IgG AF488 (Invitrogen Life Technologies, San Diego, CA, USA), and goat anti-mouse IgG AF647. DNA was stained with 4′,6-diamidino-2-phenylindole (DAPI, Sigma-Aldrich), and NETs were visualized by using a Zeiss Axioplan 2 Imaging fluorescence microscope in conjunction with a Zeiss AxioCam MRm monochromatic CCD camera and analyzed with Axiovision 4.8.2 software (Carl Zeiss). A minimum of 20 fields (at least 1,000 PMNs) per case was evaluated for MPO/NE and DNA co-staining; nuclear phenotypes and NETs were counted and expressed as percentage of the total number of cells in the fields.

### RA serum depletion, IgG purification, and quantification of NETs

After three washes with PBS, 200 μl protein G agarose (Pierce Biotechnology Inc, Rockford, IL, USA) was incubated with 200 μl ACPA + and ACPA- RA or control serum diluted in an equal volume of phenol red-free RPMI 1640 medium overnight at 4°C. The serum/protein G agarose mixture was centrifuged at 2,500 *g* for 5 minutes, and the supernatant (IgG-depleted serum) was carefully transferred into a new Eppendorf microcentrifuge tube. The protein G agarose pellet was gently washed 3 times with 500 μl ddH_2_O, and the bound antibody was released by the addition of 50 μl 0.1 *M* glycine pH 2–3, and immediately equilibrated with 10 μl of 1 *M* Tris pH 7.5-9. All protein concentrations were determined with the MN Protein Quantification Assay (Macherey-Nagel GmbH, Düren, Germany), and isolation of IgG was verified with Coomassie staining of SDS-PAGE.

Neutrophils from healthy donors (*n* = 3) were isolated and cultured for 2 hours in 96-well culture dishes (Thermo Fischer Scientific, Waltham, MA, USA), supplemented with: serum, depleted serum, and purified IgG from ACPA-positive RA patients (*n* = 3), ACPA-negative RA patients (*n* = 3), and healthy individuals (*n* = 3) to a final concentration of 100 μg/ml.

NETs were quantified after IHC staining with mouse anti-human MPO antibody (Abcam) and rabbit anti-human citH3 antibody (Abcam), or the respective isotype controls, followed by incubation with goat anti-mouse IgG AF555 and goat anti-rabbit IgG AF488 (Invitrogen Life Technologies). DNA was counterstained with 4′,6-diamidino-2-phenylindole (DAPI, Sigma-Aldrich). NETs were visualized by using an Olympus IX81 motorized epifluorescence microscope (Olympus America Inc., Center Valley, PA, USA) in conjunction with an Olympus XM10 monochromatic CCD camera (Olympus) and analyzed with the Olympus CellSens Dimension software (Olympus). A minimum of 20 fields at 10× magnification (at least 500 to 1,000 PMNs) per case was evaluated for MPO/citH3 and DNA co-staining through ImageJ analysis software (National Institutes of Health Image Processing, Bethesda, MD, USA); nuclear phenotypes and NETs were determined, counted, and expressed as percentage of the total area of cells in the fields [24].

### Protein isolation and Western blot analysis

Total protein was isolated with NucleoSpin TriPrep kit (Macherey-Nagel) from 3 × 10^6^ PMNs. Proteins from the nuclear and cytoplasmic fractions were isolated by using the Nuclear and Cytoplasmic Protein Extraction Kit (Thermo Scientific). Western blotting was performed by using AnykD Mini-PROTEAN TGX Gels (Biorad, Hercules, CA, USA) and nylon/nitrocellulose membranes (Biorad). Primary and secondary antibodies used were: rabbit anti-PAD4 (Abcam), rabbit anti-MPO (Cell Signalling Technologies, Beverly, MA, USA), mouse anti-β-Actin (Sigma-Aldrich), goat anti-Mouse and/or anti-Rabbit, human anti-HRP (Southern Biotech). HRP activity was detected by using SuperSignal West Pico Chemiluminescent Substrate (Thermo Scientific). Equal loading was verified by using beta-actin or histone H3, when appropriate. Western blots of citrullinated H3 (citH3) protein were performed according to Shechter *et al.*[25]. Densitometric analysis and protein quantification of the Western blots was performed by using the ImageJ software.

### RNA isolation and quantitative real-time PCR

Total RNA was isolated by using RNeasy Mini Kit (Qiagen). TaqMan real-time quantitative RT-PCR was performed by using the Applied Biosystems StepOne Plus cycler (Applied Biosystems) and TaqMan Gene Expression Assay primer/probe sets (Applied Biosystems) for *ELANE* (HS00236952_m1), *MPO* (HS00924296_m1), *PADI4* (HS00202612_m1), and β_2_-microglobulin *B2M* (HS99999907_m1). Data were normalized by using the housekeeping gene *B2M*, after a selection procedure involving six different endogenous reference genes, as suggested in the MIQE guidelines [26]. Relative values were calculated with 2^-DDCt^ analysis [27].

### Statistical analysis

All data are presented as mean ± SEM. Descriptive statistics for continuous parameters consisted of median and range, and categoric variables were expressed as percentages. Comparisons between patients and healthy controls were by the Mann–Whitney *U* test with a Welch posttest correction. Statistical significance in multiple comparisons was by one-way analysis of variance (ANOVA) with a Dunn posttest correction. *P* < 0.05 was considered statistically significant.

Receiver operating characteristic (ROC) curves were calculated, and the area under the curve (AUC) with corresponding standard errors of means was calculated. Data were processed in GraphPad Prism version 5.0b for MacOSX (GraphPad Software Inc., San Diego, CA, USA). Analysis of covariance (ANCOVA) was conducted with SPSS version 21.0 statistical software (IBM SPSS Inc., Chicago, IL, USA). Additional professional statistical assistance was provided by A. Schoetzau, Basel.

## Results

### RA-derived PMN exhibit increased spontaneous NETosis

Details of the RA study group and healthy control group are described in Table 1 and Additional file 1.

**Table 1:** Demographics and patient population characteristics versus healthy blood donors

	**Controls**	**RA**	**Statistics**
**Age**	50.34 ± 1.5	53.03 ± 1.5	*P* = 0.214
**Gender (F/M)**	24/32	24/8	-
**DAS28**	n.a.	3.07 ± 1.12	-
**Bone erosion (pos/neg)**	n.a.	22/10	-
**Serum ACPA (pos/neg)**	n.a.	20/12	-
**Serum RF (pos/neg)**	n.a.	19/13	-
**Serum ANA (pos/neg)**	n.a.	21/11	-
**ESR (mm/h)**	n.a.	16.8 ± 13.1	-
**CRP (mg/L)**	n.d.	6.9 ± 5.2	-
**PBMC (cells/μl)**	1,961 ± 81.69	1513 ± 75.90	*P* < 0.0001
**PMN (cells/μl)**	3,641 ± 149.7	4575 ± 546.0	*P* = 0.021
**Therapy (yes/no)**	n.a.	31/1	-
**DMARDs (yes/no)**	n.u.	27/5	-
**Biologics (yes/no)**	n.a.	30/2	-

F, female; M, male; DAS28, disease activity score; n.a., not applicable; pos, positive; neg, negative; ACPA: anti-citrullinated protein antibodies; RF, rheumatoid factor; ANA, antinuclear antibody; ESR, erythrocyte sedimentation rate; CRP, C-reactive protein; n.d., not determined; PBMC, peripheral blood mononuclear cell; PMN, polymorphonuclear leukocyte; DMARD, disease-modifying antirheumatic drug; n.u., not used.

**Figure 1:**
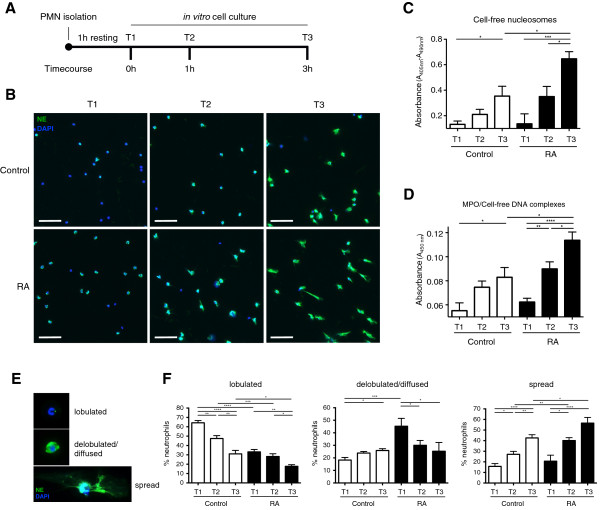
**RA-derived PMNs exhibited increased spontaneous NETosis and elevated levels of NET-component release. (A)** Schematic representation of the time-course design for studying *in vitro* spontaneous NET release. **(B)** Detection of *in vitro* NETosis by immunohistochemistry for neutrophil elastase (NE) (green) and DAPI (blue). Magnification: 20×; scale bar, 50 nm. **(C)** Concentration of cell-free nucleosomes in PMN culture supernatant by ELISA. **(D)** Quantification of NET-associated MPO/DNA complexes. These assays indicate that more-rapid and extensive progression of NET formation is observed in RA versus control PMNs. **(E)** Changes in PMN nuclear morphology during NETosis detected with immunohistochemistry for NE and DAPI. **(F)** Steady-state (T1) RA-derived PMNs exhibited a greater proportion of delobulated/diffused cells, and progressed rapidly to a NETotic spread phenotype during *in vitro* culture. Data are presented as mean ± SEM. *P < 0.05; **P < 0.01; ***P < 0.001; ****P < 0.0001. All data are representative of at least six independent experiments.

As in very recent observations [13], we observed that RA-derived PMNs underwent greater degrees of NETosis than did control PMNs *in vitro* (data not shown). To study this facet in more detail, we examined the kinetics of spontaneous NET extrusion, for which purpose, PMNs isolated from peripheral blood samples were allowed to settle for 1 hour and then cultured for a period of up to 3 hours *in vitro* (Figure 1A), NETs being detected by immunohistochemistry for neutrophil elastase (NE) and DAPI (4′,6-diamidino-2-phenylindole) (Figure 1B). In addition, we quantitatively assessed the degree of *in vitro* NETosis in these cultures by determining the concentration of cell-free nucleosomes in the respective supernatants (Figure 1C), specifically their association with myeloperoxidase (MPO), indicative of the NETotic origin of this material [2,21] (Figure 1D). We also measured NET-associated MPO enzymatic activity by using tetramethylbenzidine (TMB) as a substrate (data not shown). The results clearly indicate that RA-derived PMNs generated NETs more rapidly, to a greater magnitude, and more extensively than did control healthy PMNs, which was particularly evident at the 3-hour stage of *in vitro* culture (Figure 1B to D). Accounting for variances in PMN counts, the difference between the healthy control and RA subjects remained highly significant in an analysis of covariance (ANCOVA) of the nucleosome assay (*P* < 0.001).

During NETosis, the morphology of the PMN nucleus changes from the familiar lobulated to a diffused and then to a spread phenotype (Figure 1E) [28]. By examining and enumerating these features, it was observed that at baseline (T1), the nuclei from healthy control PMNs were predominantly lobulated, whereas in this instance, the majority of RA-derived PMN nuclei exhibited a delobulated or diffused nuclear phenotype (Figure 1F). In RA-derived PMNs, this delobulated population decreased over time, giving rise to NETotic cells with a spread phenotype (Figure 1F). In contrast, in normal PMNs, we noted a steady progression in the proportion of delobulated cells (Figure 1F). The spontaneous progression of nuclei to the NETotic-spread phenotype was more pronounced in RA than in normal PMNs, a feature most evident after 3 hours (T3) (Figure 1F).

### RA-derived PMNs demonstrate increased expression of NET-associated signaling elements, nuclear localization of PAD4, and augmented H3 citrullination

**Figure 2:**
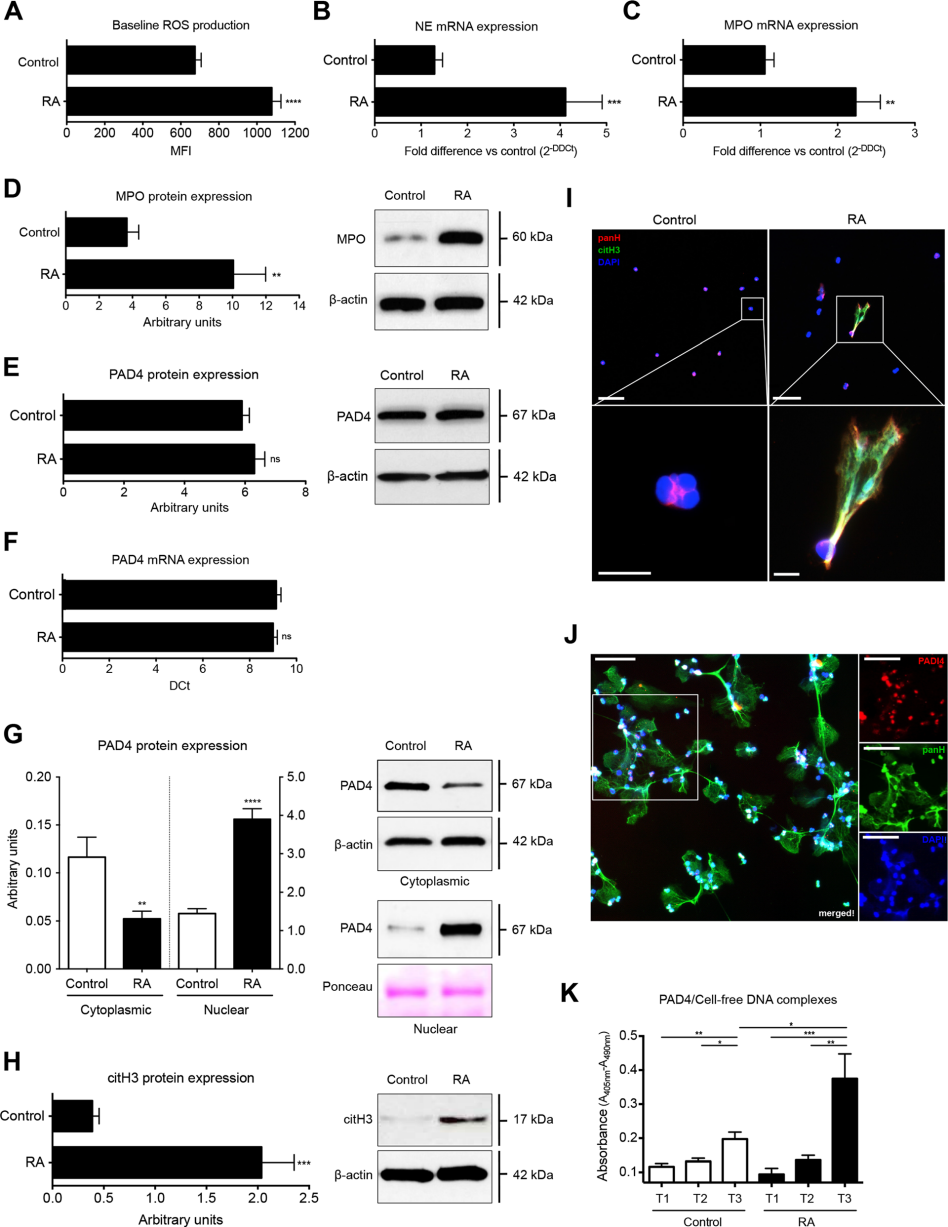
**Increased expression of NET-associated signaling elements and nuclear localization of PAD4, citrullination of histone H3 in RA-derived PMN, and possible extrusion of PAD4 on NETs.** Baseline ROS levels, measured with flow cytometry, were higher in RA-derived PMNs than in control PMNs **(A)**. Quantitative real-time PCR analysis of NE mRNA **(B)** and MPO mRNA expression **(C)**, as well as Western blot analysis of MPO protein levels **(D)**, indicated that the levels of these two components required for NETosis were elevated in RA-derived PMNs. Total PAD4 protein **(E)** or PAD4 mRNA expression levels **(F)** did not indicate any significant difference between control and RA-derived PMNs. **(G)** Quantification of PAD4 protein levels in cytoplasmic and nuclear fractions of PMNs from healthy controls and RA patients. Nuclear levels of PAD4 were significantly increased in RA patients, whereas the cytoplasmic levels were lower compared with the healthy control PMNs. **(H)** Elevated citrullinated histone H3 levels in RA PMN extracts detected with Western blot. **(I)** Co-localization of citrullinated histone H3 (green) with histone components detected with a pan-histone antibody (red), spread over the entire NET surface (blue). Magnification: upper panel 20×, scale bar, 50 nm; lower panel 63× scale bar, 10 nm. **(J)** Extracellular localization of PAD4 (red) on extruded NETs by multicolor fluorescent immunohistochemistry. NET DNA is stained blue (DAPI), and histones (panH) are stained green. Magnification, 20×; scale bar, 50 nm **(K)**. Detection of PAD4/cell-free DNA complexes in the culture supernatants of isolated PMN undergoing spontaneous NETosis. Higher levels of these complexes were detected in RA-derived PMN cultures. Time points correspond to those of Figure 1A. Data are represented as mean ± SEM. **P < 0.01; ***P < 0.001; ****P < 0.0001; n.s., statistically not significant. All data are representative of at least six independent experiments.

NETosis has been shown to depend on a number of biochemical signaling elements, among which are the generation of ROS by nicotinamide adenine dinucleotide phosphate (NADPH) oxidase, the action of NE in combination with MPO, and histone citrullination by PAD4 [2,3,5]. RA-derived PMNs exhibited increased basal intracellular ROS levels (Figure 2A), as well as increased levels of NE (Figure 2B) and MPO (Figure 2C and D), as determined with real-time PCR and/or Western blotting.

Surprisingly, neither PAD4 mRNA expression nor PAD4 levels in total cellular protein showed any discernible difference between RA and control PMNs (Figure 2E and F, respectively). Because PAD4 translocates to the nucleus on PMN activation [4,29], where it citrullinates histone proteins, such as H3, we examined its presence in nuclear and cytoplasmic PMN fractions. Compared with control PMNs, PAD4 was preferentially located in the nucleus of RA-derived PMNs (Figure 2G). The nuclear presence of PAD4 was associated with increased citrullinated histone H3 (citH3) levels with Western blot analysis in PMNs from RA cases compared with controls (Figure 2H). Furthermore, citrullinated histone H3 could be readily detected on NET structures (Figure 2I).

### Potential extracellular localization of PAD4 on NETs

Because we observed elevated nuclear translocation of PAD4 in RA PMNs, we examined whether this enzyme is extruded into the extracellular environment during NETosis. Unfortunately, the visualization of such an event by fluorescence immunohistochemistry proved to be difficult with a variety of commercially available antibodies, and we obtained only rudimentary evidence for the presence of PAD4 on NETs by this means (refer to Figure 2J).We were, however, able to detect PAD4/cell-free DNA complexes readily in culture supernatants from isolated PMNs, the levels of which were increased in cases with RA (Figure 2K). It is, therefore, quite probable that PAD4 is associated with NETs structures after aberrant NETosis in RA.

### PMNs from RA patients present an enhanced NETotic response to PMA, and normal PMNs are strongly affected by RA serum and synovial fluid

**Figure 3:**
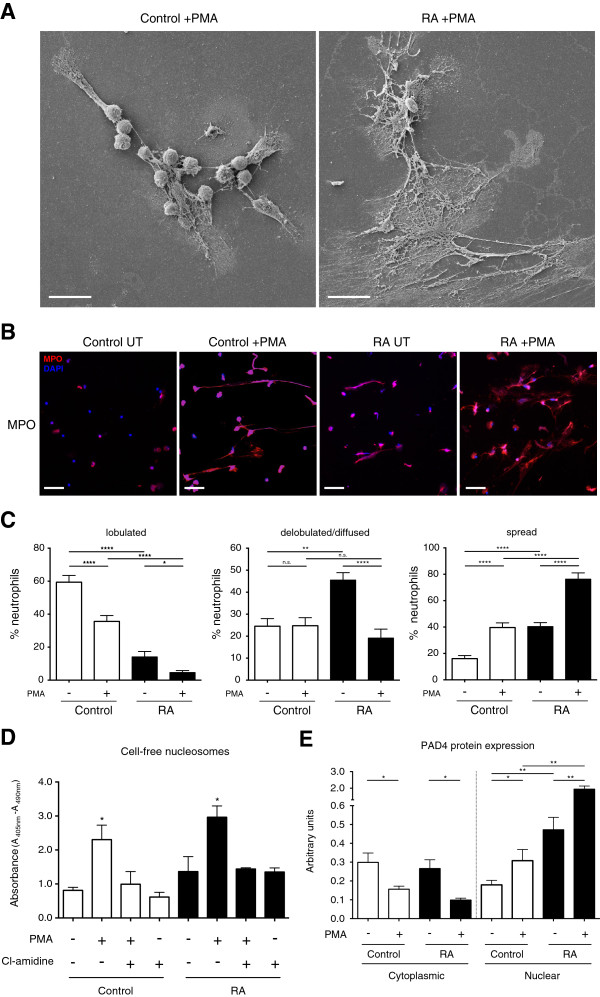
**Increased NETotic response of RA-derived PMNs to PMA. (A)** Scanning electron micrographs of NETs induced by PMA (25 n*M*) indicate the excessive NETotic response of RA-derived PMN. Scale bar, 20 nm. **(B)** Assessment of NETs induced by PMA treatment by fluorescent immunohistochemistry for MPO (red) and DAPI (blue), indicating the increased response of RA PMNs to PMA (25 n*M*). Magnification, 20×; scale bar, 50 nm. **(C)** Analysis of the nuclear phenotype indicated a vast decrease in delobulated/diffused RA PMN nuclei after treatment with PMA and rapid increase in the NETotic-spread phenotype. **(D)** Release of cell-free nucleosomes after PMA treatment is abrogated by chloramidine, a PAD4 inhibitor. **(E)** Increased nuclear localization and concomitant decrease in cytoplasmic PAD4 protein levels after PMA treatment, with a clear tendency for an increased responsiveness to the PMA stimulus by RA PMN. *P < 0.05; **P < 0.01; ***P < 0.001; ****P < 0.0001; n.s., statistically not significant. All data are representative of at least four independent experiments.

In autoinflammatory or malignant conditions, such as SLE or cancer, an elevated NETotic response of PMNs to an external activation signal has been shown [9,13]. In our experiments, we noted that when RA-derived PMNs were treated with PMA, they responded far more vigorously with regard to NETosis than did controls, as detected by SEM and fluorescence microscopy (Figure 3A and B, respectively).

In addition, morphometric assessment indicated that RA-derived PMNs exhibited a larger decrease in cells with a delobulated phenotype and a greater progression toward a NETotic-spread nuclear phenotype than control PMNs (Figure 3C), a feature accompanied by excessive release of cell-free nucleosomes in culture supernatants (Figure 3D). PMA appears to activate PAD4, as it enhanced translocation from the cytoplasm to the nucleus (Figure 3E). The stimulatory effect of PMA on the release of nucleosomes into the supernatant was abrogated by Cl-amidine, a chemical inhibitor of PAD4, indicating that PAD4 signaling is necessary for NETosis induced by PMA [4,30] (Figure 3D). These data indicate that PMNs in RA are susceptible to increased NETosis after stimulation by a secondary signal, such as that mediated by PMA.

**Figure 4:**
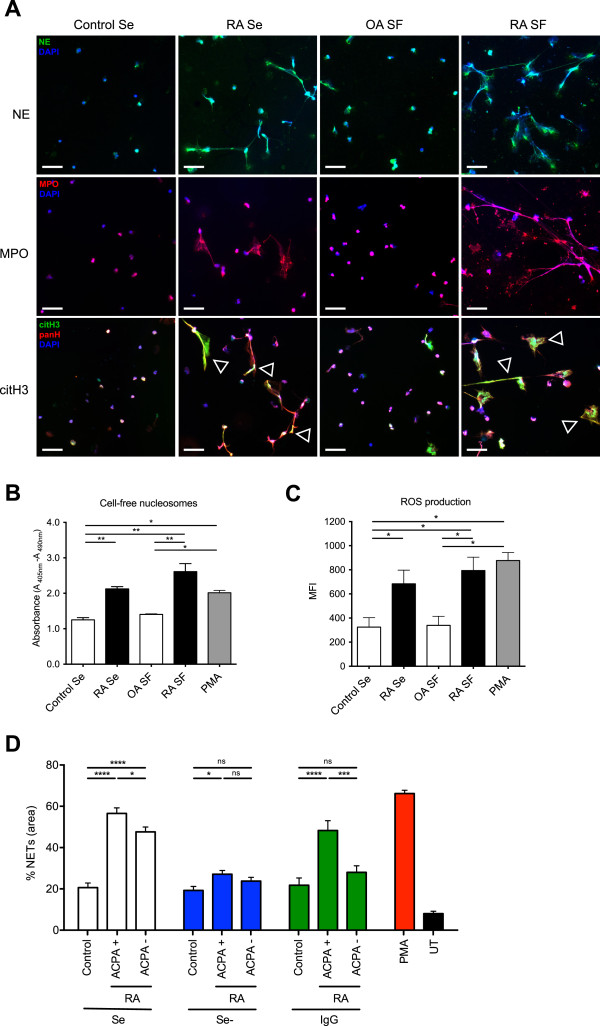
**Influence of RA serum and synovial fluid on normal PMNs. (A)** Incubation of healthy donor PMNs with serum (Se) from healthy donors or from RA patients, synovial fluid (SF) from patients with noninflammatory osteoarthritis (OA) or synovial fluid from RA patients. Immunohistochemical analysis of four main components of NETs (NE, MPO, PAD4, and citH3) revealed that RA-derived serum and SF enhanced NETosis in normal PMN compared to healthy control serum or noninflammatory OA SF. PAD4 (white arrowheads) and citH3 (empty arrowheads) colocalize with unmodified histones on NETs. Magnification, 20×; scale bars, 50 nm. **(B)** Release of cell-free nucleosomes during *in vitro* culture by PMNs from healthy controls incubated with control serum, RA serum, OA SF or RA SF, or PMA. Data are represented as mean ± SEM. *P < 0.05; **P < 0.01. **(C)** Increased ROS generation during *in vitro* culture by PMNs from healthy controls incubated with control serum, RA serum, OA SF or RA SF, or PMA. Data are presented as mean ± SEM. **(D)** Release of cell-free nucleosomes during *in vitro* culture by PMNs from healthy controls, ACPA-positive and ACPA-negative RA patients incubated with serum (Se +), IgG-depleted serum (Se -), or serum reconstituted with their respective eluted IgG, or with PMA. Data are presented as mean ± SEM. *P < 0.05; ***P < 0.001; ****P < 0.0001; n.s., statistically not significant. All data are representative of at least four independent experiments in Figures 4A–C, and 3 in 4D.

Because SLE sera and RA sera and synovial fluid (SF) have been shown to confer an increased NETotic response [9,13], we examined whether RA-derived sera or SF exerted similar effects on normal PMNs. As noninflammatory controls, we used healthy serum or osteoarthritis SF. Both RA sera and SF induced a pronounced increase in NETosis, which was paralleled by an increase in the nucleosome content of the supernatant (Figure 4A,B), as well as in ROS production (Figure 4C) when compared with healthy serum or osteoarthritis SF, respectively.To assess whether antibodies participate in the effects of RA serum on normal PMNs, we depleted IgG from serum of ACPA-positive and -negative RA patients and healthy controls. Compared with nondepleted sera, IgG depletion of both ACPA-positive and -negative sera markedly reduced NET induction to levels of normal serum (Figure 4D). Whereas the reconstitution of ACPA-negative IgG to serum did not increase NET formation significantly compared with controls, it was practically reversed to the original value in the ACPA-positive cases. This indicates a prominent role for ACPA in the induction of NETs in ACPA-positive RA, while suggesting that an alternative mechanism is responsible for the increased NETosis in ACPA-negative RA patients.

These data are in accordance with recent findings that RA-derived serum and SF induce NETosis in normal PMNs, and that ACPA and also IgM RF are to a large part responsible for this effect [13,14].

### Serum from RA patients shows elevated levels of the principal components of NETs, indicating enhanced NET extrusion during clot formation, which has potential clinical utility

**Figure 5:**
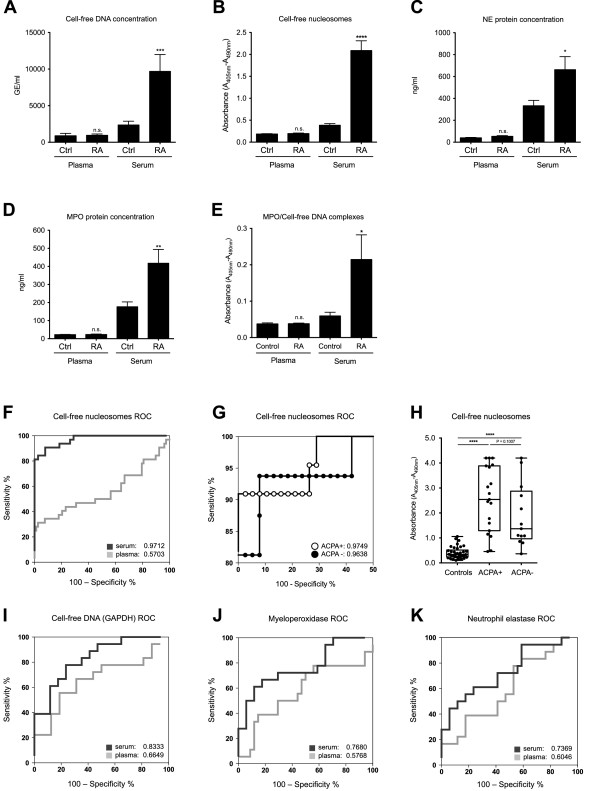
**Elevated serum levels of NET components in RA patients have potential clinical utility. (A)** Cell-free DNA levels in plasma and serum from healthy matched blood donors (*n* = 41) and patients with RA (*n* = 32) determined with real-time PCR. **(B)** Cell-free nucleosome levels in plasma and serum from healthy donor controls and patients with RA, determined with ELISA. **(C)** Determination of NE protein concentrations in plasma and serum from healthy donors and patients with RA, as assessed with sandwich ELISA. **(D)** MPO concentrations in plasma and serum from healthy donors and patients with RA, as determined with sandwich ELISA. **(E)** NET-associated MPO/DNA complexes quantified by using a modified capture ELISA. In contrast to the serum levels, none of the plasma levels of these NET components attained statistical significance (Figure 5A to E). **(F)** ROC analysis of cell-free nucleosomes in serum of patients with RA and healthy controls. **(G)** Detail of cell-free nucleosome ROC curve with groups of ACPA + and ACPA- RA cases and **(H)** scatterbox and whisker plots with individual values for control, ACPA + and ACPA- groups. The ROC curve analysis of other NET components, cell-free DNA **(I)**, NE **(J),** and MPO **(K)**, was not as conclusive as that for cell-free nucleosomes. *P < 0.05; **P < 0.01; ***P < 0.001; ****P < 0.0001; n.s., statistically not significant.

As we had previously observed increased levels of cell-free DNA (cfDNA) in RA sera [15], we determined whether this resulted from enhanced NETosis, and whether this could have diagnostic applications. cfDNA concentrations were indeed significantly higher in serum samples from RA cases compared with age-matched healthy control sera (Figure 5A). In parallel, the concentrations of cell-free nucleosomes, NE, and MPO were significantly elevated in RA serum compared with control sera (Figure 5B to D). The association of a significant fraction of MPO with markedly elevated cell-free nucleosomes in RA serum, which constitute a main component of NETs (Figure 5E), clearly suggests that NETosis is indeed the source of nucleosome material present in RA serum [21].Since there was no significant elevation of these parameters in simultaneously obtained plasma samples that were processed immediately, these data demonstrate a propensity for RA PMN to undergo increased NETosis during coagulation (Figure 5A to E).

To ascertain whether NET-associated serum components could be diagnostically useful, we conducted ROC analyses. For serum cell-free nucleosomes, this yielded the surprisingly high AUC value of 0.97 (see Additional file 2 and Figure 5F). Of interest is that no significant difference was found in this value regardless of whether the RA cases were ACPA positive or not (Figure 5G), although a slight trend for serum nucleosome levels was higher in ACPA-positive cases than in ACPA-negative cases (Figure 5H). The AUC for serum nucleosomes was significantly higher than for any of the other parameters examined (Figure 5I to K). With the cut-off set at 0.78, the ROC AUC translates into a sensitivity of 91%, with a specificity of 92% for differentiating between RA cases and healthy controls.

In contrast to RA cases, cell-free nucleosome serum values of 14 cases with SLE showed a slight, but statistically significant increase (Additional file 2). This translated into an ROC AUC of 0.7639 (see Additional file 3), which is below the clinically useful value for diagnostic purposes.

## Discussion

Although PMNs figure prominently in the joint effusions and inflamed synovial tissue of RA patients [31], the potential roles of NETotic events in the pathophysiology of this disorder have only recently become a focus of attention [13,14]. These studies indicated that RA-derived PMNs were more prone to undergo NETosis, and that NETs themselves could contribute to the generation of auto-antigens (ACPAs) or be the target of auto-antibodies [13,14].

Our studies, performed independently at a time similar time to these, corroborate that NETosis is enhanced in RA, confirming a possible fundamental role of this phenomenon in the underlying etiology of RA. In addition, we extended these observations by examining for changes in the underlying signal-transduction cascade required for the induction of NETosis. The results show that the propensity of circulatory PMNs in RA patients to undergo NETosis is associated with elevations in members of this cascade, including increased intracellular ROS production, enhanced expression of NE and MPO, increased nuclear translocation of PAD4, and citrullination of histones, notably H3. Consequently, these and other key NETotic pathway elements [6] could serve as potential therapeutic targets for interventional strategies.

Furthermore, by examining kinetic changes during extended *in vitro* culture, different nuclear morphometric characteristics were observed in PMNs from RA cases, with a lower proportion of the classic lobulated phenotype, coupled with a much higher proportion of delobulated cells at the initial time point. Unlike in controls, in which an increase in this population was noted over time, this latter population decreased during *in vitro* culture in RA PMNs. RA PMNs also progressed more rapidly and extensively to a NETotic-spread phenotype than controls, a finding confirmed by analysis of culture supernatants for the products of NETosis.

Akin to that observed in an array of other pathologic conditions ranging from preeclampsia and SLE to cancer and RA [8,9,12,13,17], PMNs from RA patients exhibited an increased response to further stimulation (for instance, by treatment with IL-8, the phorbol ester PMA, or with LPS). This response is in part mediated via the action of PAD4, as the effect of PMA could be significantly reduced by treatment with Cl-amidine, an inhibitor of PAD4 [30]. In addition, PMA treatment led to an increased nuclear localization of this enzyme, where it presumably could catalyze a more-extensive citrullination of histone proteins, thereby speeding the induction of NETosis.

Although our data are preliminary, they do suggest that PAD4 is extruded onto the NETs during NETosis, as detected with ELISA technology and, to a lesser extent, by fluorescence microscopy. Such an occurrence would have important implications for the development of anti-PAD4 autoantibodies observed in cases with RA [32]. Because the presence of such antibodies precedes the development of RA, our data provide further support that NETs may contribute to the underlying etiology of RA, and may be a relatively early event. As the presence of such anti-PAD4 antibodies has been shown to enhance the enzymatic activity of PAD4 in an extracellular environment by reducing the calcium requirement [33], their combination with NETs-associated PAD4 could lead to prodigious quantities of citrullinated autoantigens. In addition, the extracellular presence of PAD4 on NETs may further promote the prodigious generation of citrullinated antigens, because molecular structures involving the attachment of enzymes to DNA lattices have been shown to increase their catalytic activity enormously, and thereby form the basis of nano-machines or nano-factories, generating such autoantigens [34].

Although these findings must be verified, and it remains to be ascertained whether extracellular NETs-associated PAD4 is active, these data do support and extend recent reports indicating that NETs can be a source for citrullinated autoantigens, and that they react with ACPA or anti-PAD4 antibodies [13,14,35]. Taken together, these data provide further evidence concerning a key role for PAD4 in the underlying etiology of RA, and offer a potential explanation for the efficacy of PAD4 inhibitor chloramidine in reducing disease symptoms in collagen-induced rat and murine arthritis models for RA [36].

It recently was reported that ACPA or IgM RF led to potent increases in NET formation compared with control IgG [13]. In our IgG-depletion experiments on ACPA-positive and -negative RA cases, we observed a marked reduction of NET induction to control levels in both cases, whereas reconstitution of serum with IgG gained from depletion almost completely restored NET induction in the ACPA-positive cases. However, in the ACPA-negative cases, no significant increase followed reconstitution. These results support the notion that ACPAs are important inducers of NET formation in ACPA-positive RA cases, and indicate that other mechanisms, such as IgG complexes similar to those involved in NET induction in SLE [8], are operative in ACPA-negative RA. Both mechanisms could lead to a common distal mechanism of induction of arthritis.

The observation that the coagulation of blood samples from RA patients during serum preparation triggers extensive NETosis, evident by increased concentrations of cell-free DNA, nucleosomes, or nucleosome/MPO complexes, may have unexpected clinical ramifications. With a sensitivity of 91% and a specificity of 92%, it is possible that the assessment of serum cell-free nucleosomes may serve to distinguish suspected RA patients from healthy controls with a high degree of specificity. It is of interest that this aspect was not significantly influenced by the ACPA status of the RA patients. As such, this assay may be a useful complementary test to perform in conjunction with current ACPA or RF assays, not only to extend diagnostic accuracy, but also to assist in detecting RA in cases that are either ACPA or RF negative. Similar NET induction by ACPA-positive and -negative RA sera and its abrogation by IgG depletion, as discussed earlier, supports the functional aspect of the nucleosome measurement in RA serum.

In a preliminary series of SLE sera, a small and not statistically significant increase of cell-free nucleosomes over normal controls was observed, indicating a slightly elevated propensity for PMNs from SLE patients to undergo NETosis. This was, however, nowhere near the range seen in RA cases, and failed to reach an ROC AUC considered to be clinically relevant. These aspects must be validated in larger multicenter studies.

## Conclusions

In summary, our data reaffirm an intricate relation between NETosis and the etiology of RA, because the signaling elements associated with NET extrusion are significantly enhanced to promote NETosis in RA patients compared with healthy controls. Both ACPA-positive and -negative serum lost the ability to induce NETosis on depletion of IgG molecules, but reconstitution of NET induction was seen only with IgG molecules obtained from ACPA-positive serum. The assessment of NETosis-derived products in the sera of suspected RA cases may offer a novel complementary diagnostic tool.

## Abbreviations

ACPA: anti-citrullinated peptide antibody; AUC: area under the curve; citH3: citrullinated histone; DAPI: 4′,6-diamidino-2-phenylindole; DCFH-DA: 2′,7′-dichlorodihydro fluorescein diacetate; ELISA: enzyme-linked immunosorbent assay; GAPDH: glyceraldehyde-3-phosphate dehydrogenase; HBSS: Hanks balanced salt solution; MPO: myeloperoxidase; NADPH: nicotinamide adenine dinucleotide phosphate; NE: neutrophil elastase; NET: neutrophil extracellular trap; PAD4: peptidyl arginine deiminase 4; PMA: phorbol-12-myristate-13-acetate; PMN: polymorphonuclear granulocyte; RA: rheumatoid arthritis; RF: rheumatoid factor; ROC: receiver operator characteristic; ROS: reactive oxygen species; SLE: systemic lupus erythematosus.

## Competing interests

A patent filing has been submitted by the University of Basel and the Cantonal Hospital Aarau for tests developed during this research. We have no other interests to declare.

## Authors’ contributions

CSC and SG carried out molecular, cellular studies, immunoassays, and assisted with the manuscript draft. SG conducted *in vitro* depletion experiments, performed immune histochemical analyses, morphometric analyses, statistical analysis and contributed to writing the manuscript. UW and AB participated in the design of the study, assisted with stratification of patients and healthy donor controls, and assisted with the manuscript draft. SH and PH conceived the study, participated in its design, coordination and wrote the manuscript. All authors read and approved the final manuscript.

## Supplementary Material

Additional file 1: Figure S1 Neutrophil, peripheral blood leukocyte counts, and age distribution in RA cases and control cohorts. Click here for file

Additional file 2: Table S1 AUC values with corresponding 95% confidence intervals, *P* values and standard errors for serum cell-free nucleosomes and the three different parameters, which were analyzed individually by logistic regression. Click here for file

Additional file 3: Figure S2 Elevated serum levels of NET components, in RA patients, have potential clinical utility. Click here for file
