# Supplementary material for: Enhanced neutrophil extracellular trap generation in rheumatoid arthritis: analysis of underlying signal transduction pathways and potential diagnostic utility
Source: Arthritis Res Ther. 2014 Jun 13;16(3):R122. doi: 10.1186/ar4579 (PMC4229860; doi:10.1186/ar4579)
Supplement: Additional file 1: Figure S1 — Neutrophil, peripheral blood leukocyte counts, and age distribution in RA cases and control cohorts. [file ar4579-S1.pdf]

**Additional Figure 1**

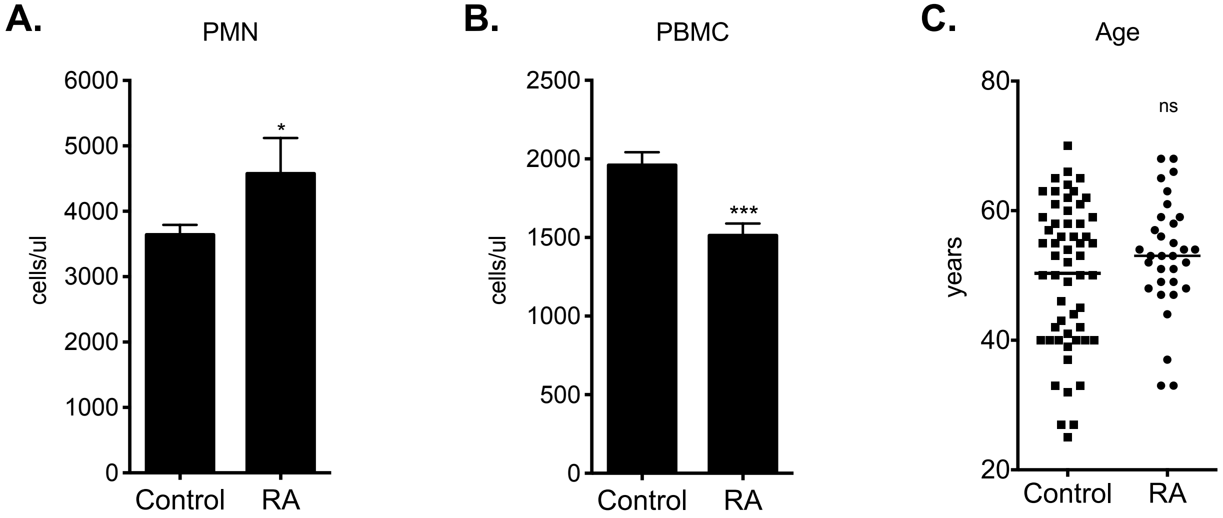


**Additional Figure 1.** Neutrophil, peripheral blood leucocyte counts and age distribution in RA cases and control cohorts.

(**A**) Neutrophil levels in cases with RA (n = 32) and matched healthy blood donors (n = 56). (**B**) Peripheral blood mononuclear cell count in RA cases and healthy control donors. (**C**) Age distribution of RA cases and matched healthy blood donors. *P < 0.05, ***P < 0.001, n.s.: statistically not significant, Mann-Whitney U test; PMN: polymorphonuclear leukocytes; PBMC: peripheral blood mononuclear cells.
