# Supplementary material for: Enhanced neutrophil extracellular trap generation in rheumatoid arthritis: analysis of underlying signal transduction pathways and potential diagnostic utility
Source: Arthritis Res Ther. 2014 Jun 13;16(3):R122. doi: 10.1186/ar4579 (PMC4229860; doi:10.1186/ar4579)
Supplement: Additional file 2: Table S1 — AUC values with corresponding 95% confidence intervals, P values and standard errors for serum cell-free nucleosomes and the three different parameters, which were analyzed individually by logistic regression. [file ar4579-S2.pdf]

Additional Table T1. AUC values with corresponding 95% confidence intervals, P values and standard errors for serum cell free nucleosomes and the 3 different parameters, which were analyzed individually by logistic regression.

Parameter	Sample	AUC	95% CI	S.E.	P value
**Cell-free nucleosomes**	Serum	0.97	0.94 to 1.00	0.016	< 0.0001
	Plasma	0.57	0.43 to 0.71	0.072	0.31
**Cell-free DNA (GAPDH)**	Serum	0.83	0.70 to 0.97	0.067	0.000
	Plasma	0.67	0.48 to 0.85	0.095	0.10
**Myeloperoxidase**	Serum	0.77	0.61 to 0.93	0.081	0.007
	Plasma	0.58	0.38 to 0.77	0.099	0.44
**Neutrophil elastase**	Serum	0.74	0.57 to 0.90	0.084	0.017
	Plasma	0.61	0.42 to 0.79	0.097	0.29

AUC: Area under the curve; 95% CI: 95% confidence interval; S.E.: standard error; GAPDH: Glyceraldehyde 3-phosphate dehydrogenase.
