# Supplementary material for: Enhanced neutrophil extracellular trap generation in rheumatoid arthritis: analysis of underlying signal transduction pathways and potential diagnostic utility
Source: Arthritis Res Ther. 2014 Jun 13;16(3):R122. doi: 10.1186/ar4579 (PMC4229860; doi:10.1186/ar4579)
Supplement: Additional file 3: Figure S2 — Elevated serum levels of NET components, in RA patients, have potential clinical utility. [file ar4579-S3.pdf]

Additional Figure 3

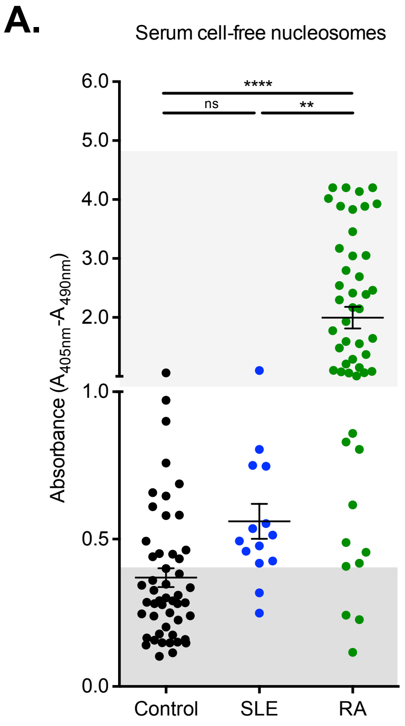

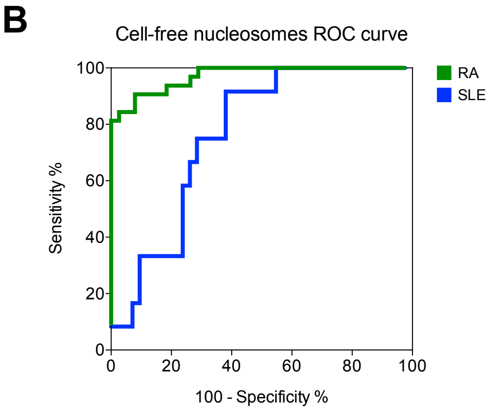

RA	Results
Area	0.9712
Std. Error	0.01598
95% confidence interval	0.9399 to 1.003
P value	< 0.0001

SLE	Results
Area	0.7639
Std. Error	0.06554
95% confidence interval	0.6354 to 0.8924
P value	0.005674

Additional Figure 3. Elevated serum levels of NETs components, in RA patients have potential clinical utility. (**A**) Cell-free nucleosome levels in plasma and serum from healthy matched blood donors (n=50), patients with SLE (n=14) and patients with RA (n=39) determined by ELISA. (**B**) ROC analysis of cell-free nucleosomes in serum of patients with RA, patients with SLE and healthy controls. **P < 0.01, ****P < 0.0001, n.s.: statistically not significant, Mann-Whitney U test; PMN: polymorphonuclear leukocytes; PBMC: peripheral blood mononuclear cells.
